# Future Fitness of Female Insect Pests in Temporally Stable and Unstable Habitats and Its Impact on Habitat Utility as Refuges for Insect Resistance Management

**DOI:** 10.1673/031.009.4401

**Published:** 2009-06-23

**Authors:** Michael A. Caprio, C. D. Parker, John C. Schneider

**Affiliations:** Department of Entomology and Plant Pathology, Mississippi State University, Mississippi State, MS 39762

## Abstract

The long-term fitness of individuals is examined in complex and temporally dynamic ecosystems. We call this multigeneration fitness measure “future fitness”. *Helicoverpa zea* (Boddie) (Lepidoptera: Noctuidae) is a polyphagous insect that feeds on many wild and cultivated hosts. While four generations of *H. zea* occur during the cropping season in the U.S. Mid Southern agroecosysem, the latter two generations were of most interest, as corn (which has been largely nontransgenic in the Mid-South) dominates the first two generations in the cropping system. In simulations of the evolution of resistance to Bt-transgenic crops, cotton refuge areas were found to be significantly more effective than similar soybean acreages at delaying the evolution of resistance. Cotton is a suitable host for *H. zea* during two late summer generations, while a soybean field is suitable for only one of these generations, therefore soybean fields of other maturity groups were simulated as being attractive during the alternative generation. A hypothetical soybean variety was tested in which a single field would be attractive over both generations and it was found to be significantly more effective at delaying resistance than simulated conventional soybean varieties. Finally, the placement of individuals emerging at the start of the 3rd (first without corn) generation was simulated in either refuge cotton, conventional soybean and the hypothetical long attractive soybean and the mean number of offspring produced was measured at the end of the season. Although females in conventional and long soybean crops had the same expected fecundity, because of differences in temporal stability of the two crops, the long soybean simulations had significantly more *H. zea* individuals at the end of the season than the conventional soybean simulations. These simulations demonstrate that the long-term fecundity associated with an individual is dependent not only on the fecundity of that individual in its current habitat, but also the temporal stability of habitats, the ecosystem at large and the likelihood that the individual's offspring will move into different habitats.

## Introduction

Insects living in complex agroecosystems may have the opportunity to oviposit in many different habitats. Some habitats may serve as sinks (net immigration, deaths exceed births), while others serve as sources (net emigration, births exceed deaths) (Pulliam and Danielson 1991). The proportion of a female's eggs that ends up in each habitat will depend on dispersal abilities of the female, host preferences and the spatial and temporal proximity of the natal habitat to other habitats. A habitat may vary in its attractiveness temporally, and a female eclosing in an adjacent field may or may not choose to oviposit in that field depending on temporal synchronicity. For example, in the Mississippi Delta, corn is planted within a relatively tight temporal window in the spring. Virtually all of the corn is unsuitable habitat for the corn earworm, *Helicoverpa zea* (Boddie) (Lepidoptera: Noctuidae), oviposition by mid-July (Snow and Brazzel 1965; Neunzig 1969; Graham et al. 1972; Stinner et al. 1976; Storer et al. 2003). As *H. zea* emerge as adults from corn, their tendency to oviposit in nearby soybean fields will depend on the flowering status of the soybean, which is in turn affected by the soybean maturity group planted and planting date (Hillhouse and Pitre 1976; Stadelbacher et al. 1984; Lambert 1988). Early maturing soybean may have completed flowering, while late planted soybean may not yet be flowering. If the alternative habitats available were cotton fields planted to varieties expressing Bt-toxins (sink habitats), a given female might lay the same number of eggs, but the proportion of those eggs surviving to adulthood would vary with the maturity status of the soybean and the proportion of the eggs laid on transgenic cotton. More importantly, the proportion of eggs subjected to selection for Bt resistance depends on the maturity status of soybean.

Most measures of fecundity examine the reproductive output of a female on a given host plant or in one habitat. In general, the number of eggs a female can expect to oviposit is examined, ignoring the environments in which she is likely to oviposit. We suggest that in complex landscapes, an additional measure of fecundity is needed, a measure not only of a female's reproductive output, but also of the reproductive output of her offspring, and perhaps their offspring as well. We call this measure “future fitness”. This measure is needed because, while two habitats might be equally suitable hosts, differences in their temporal stability, vicinity to sink habitats and other factors might impact the survivorship and fecundity of the offspring produced. The habitat chosen by a female might not impact the number of F1offspring, but it could impact the F_2_ or F_3_ generations through landscape-scale interactions (Caprio 2001).

While closely linked to the theory of source-sink dynamics, future fitness is a measure of the long-term population growth associated with a given female as she, and her offspring, move throughout the ecosystem. Sourcesink theory describes the ecosystem at a habitat level, while future fitness is an estimate of the impact and interaction of those habitats on the long-term fitness of an individual and her offspring.

In the mid-southern U.S., *H. zea* utilizes a large number of crop and non-crop hosts over the course of the spring, summer and fall. In the early spring it is found on several wild hosts such as geranium and crimson clover (Stadelbacher et al. 1986). Corn becomes available as a host in June and is highly attractive to the ensuing two generations. Following the dry-down of corn in mid-July, *H. zea* moves into a complex of crops including cotton, soybean and sorghum, spending 1 to 2 generations in these crops before entering diapause or moving on to wild hosts (Parker 2000). The cotton may be further divided into transgenic insecticidal cotton, refuge cotton that is not sprayed, and conventional cotton which is sprayed. The latter includes refuges for transgenic cotton using a 20% refuge option. Soybean and sorghum may vary in their planting date, and soybean may further vary by maturity group. It has been suggested that these two crops might serve as functional refuges for transgenic cotton varieties, perhaps justifying relaxation of the cotton refuge requirements mandated by the U.S. Environmental Protection Agency. The appropriateness of these alternative hosts as refuges is dependent on a number of issues, including life history traits in each habitat as well as dispersal and future fitness of individuals ovipositing in them.

In order for a habitat to be effective as a refuge and delay resistance, it must serve two functions. It must produce enough individuals to mate with potential resistant individuals that emerge in transgenic fields. In addition, the refuges must repopulate themselves, i.e., the net reproductive rate in refuges should be ≥1.0, at least averaged over the entire year.

Another situation where future fitness may be relevant is the use of seed-mixtures of transgenic and non-transgenic cultivars. Mallet and Porter (1992) suggested that larval movement among transgenic and non-transgenic plants would alter the effective dominance of the resistance trait and hasten the evolution of resistance. However, regardless of the impact of larval movement, seed-mixtures that are at the low end, and large continuous refuges at the high end of a spectrum of refuge isolation can also be considered (Caprio 2001; Caprio et al. 2004). The future fitness of a female ovipositing in the contiguous nontransgenic cotton would be greater than a female ovipositing in a mixed field because her offspring are more likely to oviposit on non-transgenic cotton.

We propose the following application of the concept of future fitness to *H. zea*. The future fitness of an individual moving from corn into an untreated cotton refuge is greater than that of an individual moving from corn into a soybean field. This follows from the fact that while soybean fields in general may be present as refuges for several generations of *H. zea*, any one field is only suitable for a single generation after which future generations must find new habitats chosen at random from those available. Specifically, we predict that changing a simulated agroecosystem so that individual soybean fields remain attractive for 6–8 weeks (while maintaining the same proportion of attractive soybean fields across the season) will decrease the rate of resistance evolution to Bt-cotton by increasing the future fitness of females moving into this habitat.

There is considerable debate about the production of *H. zea* from soybean relative to cotton during the latter part of the season, but this is irrelevant to the hypothesis. The difference that is relevant is that a field of cotton is an attractive and suitable host plant throughout the latter half of the season, spanning at least two generations of *H. zea*, while a soybean field is at best only attractive and suitable for a single generation.

## Materials and Methods

### Simplified cotton-soybean simulations

The cotton-soybean system simulated for *H. zea* was based on the ILSI-HESI model (ILSI-HESI 1999) and consisted of 1024 patches arranged in a 32 × 32 matrix. Patches are areas of uniform habitat and multiple patches may make up a single field. While the original model incorporated habitats such as corn and sorghum (Table 1), The model was simplified to 8 habitats, early and late soybean, transgenic Bt-cotton, conventional cotton and Bt and non-Bt corn, early season geranium and early season clover. The simulation included 3 levels of soybeans (0, 20 and 40 patches) with 3 levels of refuge cotton (0, 20 and 40 patches). The differences between the levels of soybean and cotton were analyzed using 2-way ANOVA. Fallow patches were used with diminishing levels of refuge cotton and soybean to maintain the same overall number of patches. There were 360 patches of Bt-cotton. 500 patches of corn habitat were equally divided among Bt-corn (assumed to express the same trait expressed in Bt-cotton) and non-Bt corn. 60 patches were early season geranium and 24 were early season clover. Habitat suitability was dynamic over the course of a season (Table 2). Monogenic resistance was assumed, and the mortality rates (in addition to natural mortality) on transgenic cotton for susceptible, heterozygous and resistant individuals were 96%, 92% and 5%, respectively. High overwintering mortality (95%) (Caron et al. 1978; Stadelbacher et al. 1986) and overwintering dispersal (90% of overwintering individuals were randomly assigned new fields during overwintering) were included in the model. The latter is justified because moths move out of cotton and spend 2 generations on alternative hosts prior to re-entering the corn-cotton agroecosystem in the late spring.

**Table 1.**
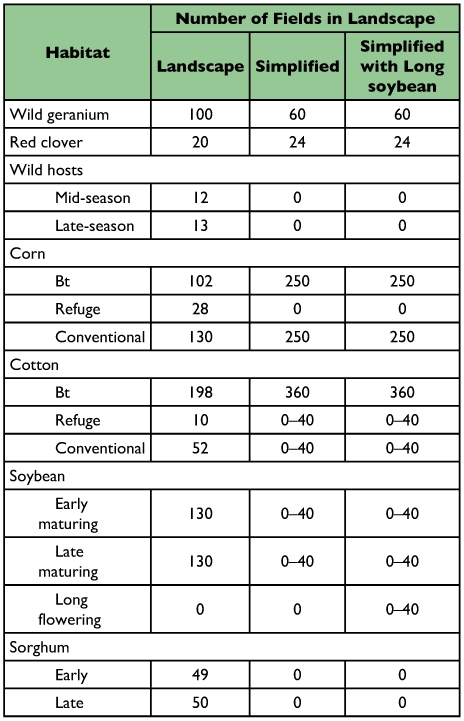
Simulated fields (each representing 100 acres) planted to different habitats. Individual fields were randomly distributed through the system.

During the course of the experiment, non-linearities were noted in response to changes in number of cotton refuge patches. To understand the cause of these non-linear responses and to determine the impact of Bt-corn on this system, an additional set of simulations was done with no soybean patches, 500 non-Bt-corn patches (and 0 Bt-corn patches) and the number cotton refuge patches was varied over 3 levels (0, 20 and 40 patches).

**Figure 1.**
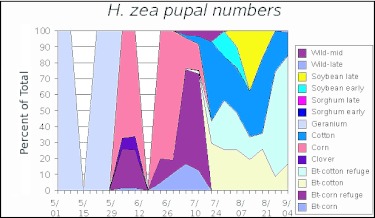
The proportion of simulated *Heliothis zea* pupal numbers in different habitats throughout a simulated growing season in the Mid-South. The approximate ends of generations are 5/15 for GO (on wild host plants), 6/15 for G1, 7/15 for G2, 8/15 for G3 and 9/15 for G4. Note that the simulation stops slightly early for G4 because these individuals overwinter as pupae and larvae present at 9/4 are assumed to overwinter as well. While the first two generations are distinct, they begin to overlap by G3.

**Figure 2.**
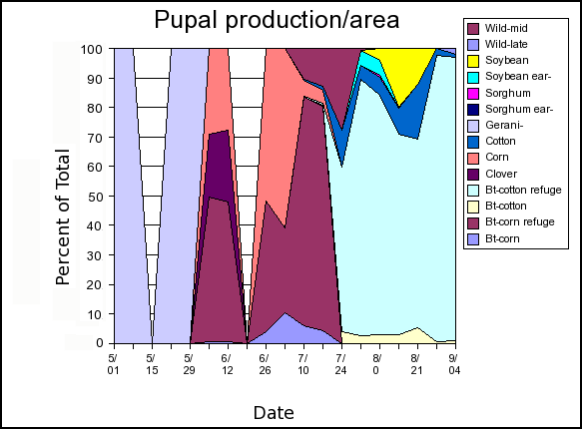
Pupal production per unit area in the spatially complex landscape model of *Heliothis zea*. The generations are as described in Figure 1.

Landscape simulations. A second model was developed that incorporated additional information about the agroecosystem used by *H. zea* in the Mid-South. This model included 14 different habitats (Table 1) that were dynamic over the season (Table 2). No single, consistent study has been conducted to compare life tables from the various habitats, so the values of life table variables for the 11 different habitats included in the model (Table 3) were based on a variety of sources, including published work and input from researchers, extension personnel and grower consultants (Henry and Adkisson 1965; Snow and Brazzel 1965; Lindgren et al. 1968; Johnson et al. 1975; Gross and Young 1977; Roach and Ray 1976; Slosser et al. 1978; Pitre and Hillhouse 1981; Hogg and Nordheim 1983; Stadelbacher et al. 1986; Kring et al. 1993; Neussly et al. 1994; Sudbrink and Grant 1995; Peck et al. 1999; Parker 2000; Sansone and Smith 2001; Storer et al. 2001; Han and Caprio 2002; Gore et al. 2003). Mating was assumed to be random within a patch. *ON*, the proportion of oviposition in the natal habitat, was estimated based on the fecundity, mortality and dispersal rates entered into the model. Carrying capacity reflects the maximal populations that might exist in each habitat in the absence of any control measures. Highly managed crop systems rarely approach these limits. *Bt*-habitats are not shown as they were identical to non-*Bt*-habitats except for genotype-specific larval mortality as described previously. Figure 1 shows the densities of pupae corrected for the area of each crop. This figure reflects the numbers of individuals actually contributed to future generations by each host and describes the importance of the hosts in the evolution of resistance. The proportion of the pupal densities in each habitat as a function of time (Figure 2) were a reasonable description of observed densities on known hosts (Parker 2000). This figure reflects the relative numbers that would be observed when sampling individual hosts. Other, undetermined, hosts or immigration from other areas could alter the distribution of adult densities (Gould et al. 2002; Gustafson et al. 2006).

**Table 2.**
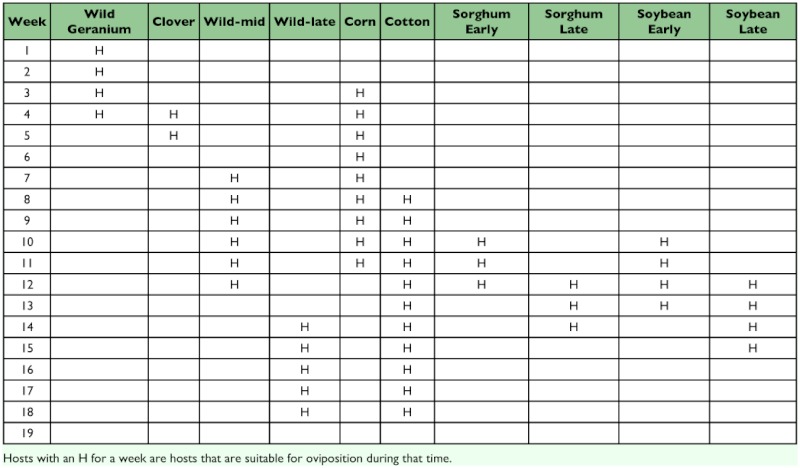
Suitability of habitats for Heliothis zea oviposition over the summer season. Larval populations continue to develop normally after ovipositional suitability has declined.

This model did not include directed movement by adults to suitable habitats (Stinner et al. 1974, Stinner et al. 1976), but rather depended upon migration rates out of habitats. Habitats suitable for oviposition had relatively low dispersal rates, while unsuitable hosts had high rates of dispersal (95%/day). Thus the primary mode of population redistribution was by dispersal out of unsuitable habitats and retention of adults in more suitable habitats. While plants might be unsuitable for adult oviposition, larvae and pupae already in those fields would continue to develop normally and the resulting adults disperse at high rates.

Individual movement was modeled as a two step process. First a draw was made from a binomial distribution to determine the number of individuals that would leave a patch. The probability of dispersal varied with the habitat and the temporal condition of the crop (Tables 2, 3). For each dispersing individual, two draws were made from a precalculated table of dispersal distances using a single-dimension diffusion equation with a diffusion constant of 64 (Rudd and Gandour 1985). The results of these draws represented dispersal distances in the ×and y directions, and adding these two vectors resulted in the final dispersal vector for that individual. A torus construction was assumed so that individuals dispersing off the grid looped around to the opposite side.

### Long soybean simulations

To determine if the temporal instability of the soybean habitat was contributing to a reduction in the habitat's capability to delay resistance to transgenic crops, the dynamics of the ecosystem was modified so that early soybean remained an attractive habitat from day 50 to day 91 (the period normally covered by both early and late soybean). The number of attractive soybean fields remained at 40, and the simulations otherwise used parameters from the standard landscape simulations. Comparisons were made on 20 runs with no soybean fields, 20 runs with 40 early and 40 late soybean fields, and finally 20 runs with 40 long soybean fields using one-way analysis of variance, and multiple comparisons were done using a Bonferroni-adjusted alpha level of 1%.

**Table 3.**
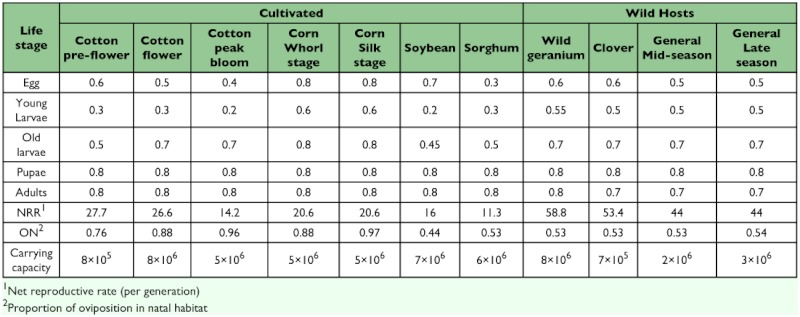
Crop and stage specific survivorship rates for the major habitat types included in the model. Daily survivorship rates depend on the length of the stage.

### Future fitness simulations

To test the prediction that female future fitness is impacted by landscape scale interactions, three additional sets of simulations were done, each starting on day 78, the day when corn is no longer a suitable host for oviposition in the model. Each set of simulations started with no individuals in the system on day 78 and one habitat that was populated with 2,000 pupae/field. The habitat initially populated with pupae was refuge cotton, early soybean or long soybean. At the end of the season (day 133) all individuals (including overwintering pupae) present in the entire simulated system were censused. Because only one habitat was initially populated with pupae, the total population at the end of the season represented the future fitness of the initial females after 55 days. Census numbers were corrected for number of original female pupae as there were unequal numbers of refuge cotton and soybean habitats. Five replicate simulations were run for each habitat and the results were analyzed with ANOVA.

## Results and Discussion

### Simplified Cotton-Soybean simulations

**Figure 3.**
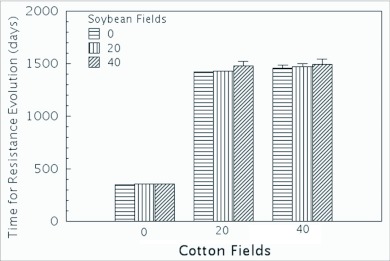
Response (mean time to resistance) to changes in levels of cotton refuge and soybean fields. The surface is the linear least-squares fit to the data.

Changes in soybean acreage configuration had no significant impact on time until resistance evolved (Figure 3). The main effect of the soybean level was not significant in the ANOVA (*f* = 0.1237; *df* = 1, 42; *p* = 0.7268). Resistance times only decreased significantly when the cotton refuge was reduced {*f* = 138.9387; *df*= 1, 42; *p* <0.001). These data suggest that the soybean fields, though relatively productive on a per generation basis, were not as effective as conventional cotton refuges at delaying resistance. The primary difference between the non-transgenic cotton fields and the soybean fields in this simulation was the temporal stability of the cotton fields. Cotton refuge fields were suitable habitat through both generations in the latter half of the season (but not during overwintering), while any given soybean field was suitable only for a single generation.

**Figure 4.**
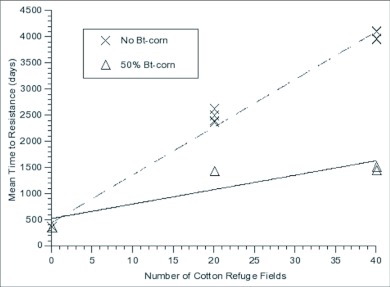
Comparison of simulations with and without Bt-corn. No soybean refuges were included.

The mean time to resistance did not significantly change between the two upper levels of cotton refuge (20 and 40 patches). These correspond to 5% and 10% of the total number of cotton patches. The lack of response in time to resistance to this change could be due to non-linearities in response to the level of refuge, or it could be due to selection in the corn in the first half of the season predominating and masking selection in the latter half of the year. The latter proposal is supported by the results from the runs without Bt-corn in the system (Figure 4). When Bt-corn was absent from the system, increasing refuge areas in cotton significantly increased the time it took for resistance to evolve at all levels. These results suggest that our ability to predict the impact of refuges in one crop is dependent on the overall landscape. In order to effectively manage resistance for polyphagous pests, the landscape as a whole needs to be assessed instead of focussing on individual crops (Caprio 1994; Kennedy and Storer 2000).

**Figure 5.**
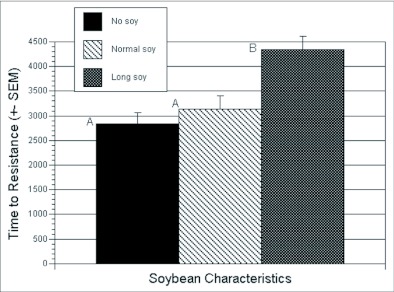
Changes in the time required for the evolution of resistance for two different phenologies of the soybean crop.

### Landscape simulations

The proportions of all pupae in each habitat for the artificial landscape are shown at one week intervals in Figure 1. The relative densities of pupae per unit area in each habitat are given in Figure 2. The greatest density of *H. zea* pupae was found in the cotton refuge habitat. Lower densities were found in conventional cotton because insecticidal sprays are applied to these areas (hence the U.S. EPA requirement in the U.S. for a 4-fold larger refuge when refuges are sprayed and transgenic fields are not). The simulation output agrees qualitatively with landscape utilization by *H. zea* in the mid-Southern U.S., though there are some discrepancies. Gustafson et al (2006) estimated that adult *H. zea* production per hectare from soybean fields in Mississippii was approximately 10% of the production from unsprayed non-transgenic cotton fields, approximately the rate resulting from this model for the 4th but not 3rd generations (Figure 2). Gould et al. (2002) found larger numbers of *H. zea* that had fed on C4 plants (e.g., sorghum and corn) in the latter two generations of the year than suggested by the model. Unfortunately, that method does not distinguish between migrants and locally produced moths, and it is difficult to determine the origin of these moths. Although we included an area equal to 10% of the cotton habitat as wild hosts, the populations in these fields did not produce 10% of the adult population in the latter half of the season, particularly the later (4th generation) wild host fields.

This suggests that if a wild host is responsible for the C_4_ moths identified by Gould et al. (2002), it is most likely that this would be a host that would extend over the entire second half of the growing season, limiting the impact of habitat instability on these hosts.

### Long soybean simulations

When the mean time for resistance to evolve was compared between no soybean, early and late soybean, and hypothetical long fruiting soybean, only the hypothetical long fruiting soybeans delayed resistance (Figure 5). These results agree with the simpler model that the effectiveness of soybean as a refuge is limited. They also demonstrate specifically that it is the stability of the habitat (comparing long soybean versus soybean) that is responsible for a large part of the poor performance of soybean as a refuge. Furthermore, the effect of habitat stability is not masked by other habitats in this complex landscape (transgenic cotton, cotton refuges, conventional cotton, sorghum, and wild hosts are all present at the same time of year). This suggests that the effect of stability is not small relative to other factors in this agroecosystem.

### Future Fitness

At the end of the year, there was an average of 12.12 (± 1.33) individuals in the entire ecosystem for each adult that was placed on refuge cotton compared to 3.07 (± 0.11) individuals for each individual placed on early soybean. When soybean was assumed to be long season and the same soybean fields were suitable for oviposition throughout the latter half of the season, the future fitness increased to 5.46 (± 0.53). A one way analysis of variance showed significant differences among these means (*F* = 128.1; *df* = 2,12; *P* <0.001) and a Bonferroni-adjusted comparison of the individual means indicated significant differences between all pairs of means at an alpha level of 1%. These data suggest that the future fitness of an individual female on refuge cotton in the mid-season is approximately 3.9 fold higher than the future fitness of a female on soybean. Some of this difference might be due to differences in life tables for the two crops, but the long soybean simulations, which use the soybean life table but remove the temporal instability, clearly show that temporal instability in the soybean habitat limit the ability of this host to act as a refuge. The difference in future fitness suggests a mechanism to explain why the cotton refuges may be more effective than the soybean refuges. When adult moths emerge in cotton refuges, they emerge in suitable habitat (because these refuges are stable and suitable across the latter portion of the season). Females tend to lay a disproportionate number of eggs in their natal habitat if it is still suitable for oviposition, and the future fitness of these females will be increased because their ovipositional patterns result in a bias towards eggs laid in a relatively benign habitat (though natural mortality still exceeds 95%). In contrast, although females that move into early soybean fields will find a suitable host for oviposition, when their offspring emerge from pupation the habitat will no longer be suitable and these moths will have to disperse before initiating oviposition. Their eggs are therefore likely to be laid at random throughout the suitable crops in the agroecosystem, of which a large proportion is the relatively toxic transgenic crop. This impacts both population dynamics, but also exposes a greater fraction of the total population to selection and consequently increases the rate of evolution of resistance. Even though a large number of individuals may be produced in soybean fields, because there is no bias towards these refuges repopulating themselves, they tend to be less effective than the temporally stable cotton refuges. Most of the individuals that oviposit in late soybean fields are unlikely to have originated in soybean fields, while a greater proportion of 4th generation *H. zea* on refuge cotton are progeny of individuals from this cotton habitat. In the framework of source-sink dynamics, Caprio (2001) showed that some isolation between refuges and transgenic fields could increase the effectiveness of refuges. Similarly, the temporal instability of the soybean habitat, as examined in the current model, may be thought of as effectively decreasing habitat isolation. Temporal instability leads to increased movement between habitats and greater exposure of susceptible insects to sink habitats such as transgenic cotton. As long as there is sufficient movement to ensure that resistant individuals selected for in transgenic habitats are most likely to mate with susceptible individuals from refuges, excess movement above this value is likely to increase the rate of resistance development.

The results of this paper demonstrate that, although two individuals in different habitats may have identical current fitnesses by all relevant measures, they may still have significantly different contributions to future generations. While the number and viability of F1 offspring might be identical, the relationship of their current habitat to the larger landscape can cause the sizes of the F_2_/F_3_ generations to differ in a consistent manner. In other words, two adults might have identical fitnesses by every measure in the current generation but have significantly different contributions to future generations because of interactions with the landscape at large. In the specific case of resistance management, these results suggest that habitats such as soybean are likely to be less effective as refuges because of their temporal instability relative to other potential refuges such as cotton. We suggest that many wild hosts, which tend to be short lived, will also be less effective as sources of wild, unselected individuals. Other more long-lived wild plants (e.g., velvetleaf, *Abutilon theophrasti* Medik.) might be more effective as potential natural refuge hosts.
